# The Role of Galectin-3 and ST2 in Cardiology: A Short Review

**DOI:** 10.3390/biom11081167

**Published:** 2021-08-07

**Authors:** Ana Merino-Merino, Jeronimo Gonzalez-Bernal, Dario Fernandez-Zoppino, Ruth Saez-Maleta, Jose-Angel Perez-Rivera

**Affiliations:** 1 Department of Cardiology, University Hospital of Burgos, 09005 Burgos, Spain; anamerinomerino@gmail.com; 2 Department of Health Sciences, University of Burgos, 09005 Burgos, Spain; jejavier@ubu.es (J.G.-B.); dfx@ubu.es (D.F.-Z.); 3 National Scientific and Technical Research Council (CONICET), 2290 Buenos Aires, Argentina; 4 Department Clinical Analysis, University Hospital of Burgos, 09005 Burgos, Spain; rsaezu@saludcastillayleon.es

**Keywords:** ST2, galectin-3, biomarkers, cardiology

## Abstract

Galectin-3 is a lectin that binds beta-galactosides. It is involved in cardiac remodeling and fibrosis through the activation of macrophages and fibroblasts. ST2 is secreted by myocardial cells due to cardiac overload. These two biomarkers have been traditionally studied in the field of heart failure to guide medical therapy and detect the progression of the disease. Nevertheless, there are novel evidences that connect galectin-3 and ST2 with coronary heart disease and, specifically, with atrial fibrillation. The aim of this article is to concisely review the diagnostic and prognostic role of galectin-3 and ST2 in different cardiac diseases.

## 1. Introduction

Biomarkers are molecules used as indicators of biological processes that can be objectively observed in a sample. In most cases, these are obtained from peripheral blood. Biomarkers are commonly used in clinical practice both in the diagnosis and assessment of the response to treatment of a variety of cardiovascular diseases. There are biomarkers that have been widely studied, validated, and used in cardiology, such as NT-proBNP or troponins, and other more novel and not yet well studied, such as Galectin-3 (Gal3) and ST2. The objective of this review is the analysis of these two biomarkers in the main heart diseases.

## 2. ST2 and Galectin-3: Basic Concepts

Gal3 is a protein that belongs to the family of galectins, which are beta-galactoside binding proteins. Gal3 is broadly expressed in tissues, including all types of immune cells, epithelial cells, and sensory neurons [1]. Furthermore, it participates in a wide variety of processes involved in the genesis of fibrosis, such as apoptosis, angiogenesis, and inflammation. In addition, it can regulate the effects of other molecules involved in fibrosis processes, such as cytokines, by retaining their receptors on the surface membrane of the atrial myofibroblast. In normal mouse myocardium, Gal3 was constitutively expressed in macrophages and was localized in atrial but not ventricular cardiomyocytes. It is postulated that it may be a marker in the initial phase of the fibrosis and remodeling process [2].

Furthermore, Gal3 has been previously correlated with a large number of cardiovascular risk factors [3] and has the ability to bind to the von Willebrand factor, thus being implicated in the modulation of thrombus formation in its early phase [4].

Apart from its increase in cardiovascular diseases, an elevation of Gal3 has been observed in patients with both acute and chronic renal failure. It has also been observed that Gal3 could be involved in the pathogenesis of renal cell carcinoma in men, in addition to other types of cancer, such as papillary thyroid carcinoma, hepatocellular carcinoma, prostate cancer, or pancreatic carcinoma [4].

The performance of endomyocardial biopsies in patients with heart failure (HF) showed that the concentrations of Gal3 in the cardiac tissue did not show the concentrations in plasma. In many cases, the level of Gal3 in the blood of patients with HF who underwent heart transplantation was unchanged; it is thought that other organs could be responsible for this increase in its expression [5].

The reference intervals for Gal3 in healthy individuals have been obtained from studies such as that of Framingham. In this cohort, Gal-3 concentrations were higher in women compared with men (*p* < 0.05), with median Gal-3 in women of 14.3 ng/mL (Quartile [Q] 1-Q3 12.0–16.8) versus 13.1 ng/mL (11.1–15.4) in men [6]. The value of 17.8 ng/mL, the 90th percentile of normality, is considered the cut-off value of Gal3 [7].

ST2 is a protein that is part of the interleukin-1 receptor, and there are two isoforms, a transmembrane receptor (ST2L) and a soluble receptor (sST2, denoted as ST2). Interleukin-33 plays a cardioprotective role. It prevents fibrosis and cardiac hypertrophy through ST2L. In addition, ST2 reduces the cardioprotective effect of the Interleukin-33/ST2L pathway by binding to free interleukin-33 [8]. Furthermore, ST2 is overexpressed under conditions of myocardial stress or injury and is associated with inflammation and immune response. In addition, it is thought that ST2 is secreted by the vascular endothelium and by myocardial cells because of cardiac overload [9], with the vascular endothelium expressing the greatest amount.

ST2 has been shown to have a great prognostic value in cardiovascular diseases and also in other diseases, such as chronic kidney failure. This allows a better risk assessment in end-stage dialysis patients [10]. In addition, the role of the Interleukin-33/ST2 pathway in the development of fibrosis in systemic sclerosis and other fibrotic diseases has also been studied [11]. The normal value of ST2 in healthy patients is less than 35 ng/mL [8].

The genotyping of biomarkers has not been studied. In the literature, the most studied polymorphisms related to plasmatic levels of Gal3 are rs2274273 and rs4652 [12], and of ST2, rs1558648 and rs13019803 [13]. The gene encoding Gal3 (LGALS3: “lectin galactoside-binding soluble 3 gene”) is located on chromosome 14 (14q22.3). The expression of this gene is affected by the presence of several polymorphisms, such as rs4652, which is found in exon 3, position 292, where a missense mutation due to a substitution of the nucleotide adenine (A) by cytosine (C) leads to an irregular expression by Gal3. Along with this polymorphism is rs2274273, which implies the exchange of C for thymine (T). The IL1RL1 gene encodes ST2 and is located on chromosome 2q12. The change from C to T in the rs13019803 polymorphism in the intronic region of IL1R1 on chromosome 2 and the change from A to C in the rs1558648 polymorphism in the same region and chromosome but in the IL1RL2 gene are related to the secretion of ST2 [13].

In this sense, in a study carried out by our group in patients with permanent atrial fibrillation (AF) who underwent electrical cardioversion, the plasma levels of Gal3 and ST2 were determined at baseline prior to cardioversion and at the 6-month follow-up. It was observed that the carriers of the mutated allele of the Gal3 polymorphism rs2274273 had lower levels of Gal3, measured both at baseline and the 6-month follow-up. Those patients carrying the mutated allele of the ST2 polymorphism rs1558648 had lower ST2 values measured at baseline but not at the 6-month follow-up. As a conclusion, the identification of genetic predisposition to present higher levels of harmful biomarkers might facilitate the individualization of the diagnosis or the therapeutic strategy [14].

## 3. ST2 and Galectin-3 in Ischemic Heart Disease

There are conflicting studies regarding Gal3 expression levels in acute myocardial infarction (AMI). Higher Gal3 values have been identified in patients with AMI than in patients with unstable angina and, in turn, higher values than in stable angina. Furthermore, a higher expression of Gal3 has been observed in multivessel coronary artery disease [15]. Likewise, in high cardiovascular risk patients referred for coronary angiography, Gal-3 is a strong independent predictor of cardiovascular death [16].

Patients with AMI who present higher expression of ST2 have been associated with a greater probability of cardiovascular death and HF. ST2 levels provide not only prognostic information independent of traditional risk factors, but it is also complementary to NT-proBNP, and its combination offers better risk stratification compared to the TIMI risk scale [17]. In a recent article by Aleksova et al. [8], an algorithm was proposed in AMI (type 1 and type 2) in which ST2 values <35 ng/mL translate to the absence of activation of fibrosis cascades, with which adverse remodeling is unlikely, levels between 35–70 ng/mL translate to moderate activation of fibrosis cascades and adverse remodeling is likely, and finally, ST2 levels >70 ng/mL translate to the activation of fibrosis mechanisms and neurohormonal activation. By determining these biomarkers, those patients with a higher risk of adverse left ventricular remodeling could be identified in order to adapt the management of these patients.

## 4. ST2 and Galectin-3 in Heart Failure

Gal3 has been shown to be useful in predicting the development of HF in the general population. In a Framingham Offspring study that included 3353 patients, Gal3 was associated with an increased risk of developing HF and all-cause mortality [6]. In another study that included 5958 patients, an increase in the serum levels of Gal3 was independently associated with an increased risk of developing HF [7].

In a study that included patients who were admitted for dyspnea, a cut-off value of Gal3 of 9.2 ng/mL was suggested to distinguish acute HF from other causes of dyspnea. Furthermore, the patients who died during the follow-up presented higher values of Gal3 than those who survived (12.9 vs. 9.0 ng/mL, *p* < 0.001) [18]. In the GALA study (GALectin-3 in Acute heart failure) that included 115 patients with acute HF, the values of three biomarkers, Gal3, NT-proBNP, and troponin I, were compared in the prediction of all-cause mortality at 30 days and at the 1-year follow up. This study concluded that only Gal3 was useful for predicting all-cause mortality 30 days after hospital admission for acute HF, although it had no prognostic usefulness for mortality at one year, which did show NT-proBNP. In contrast, troponin I was not useful in predicting mortality at 30 days or one year after admission [19]. In the COACH study (Coordinating Study Evaluating Outcomes of Advising and Counseling in Heart Failure), Gal3 was a highly useful biomarker in predicting the absence of events within 180 days after hospital discharge due to acute HF. It also presented a high sensitivity with values below 11.8 ng/mL and higher values (>17.8 ng/mL) have been related to an increased risk of new hospitalization for HF (up to 2–3 times higher) [20].

In contrast, in other studies, Gal3 has shown limited utility. In the RELAX-AHF (RELAXin in Acute Heart Failure) study that included 1161 patients with acute HF in whom Gal3 values were determined at different times, Gal3 values were stable over time, so no usefulness was established in event prediction [21]. Gal3 appears to be an additional risk biomarker in acute HF. However, it is not a specific cardiac biomarker, as it performs many other systemic functions, which may explain the contradictory results.

In the Val-HeFT study (Valsartan Heart Failure Trial), which included 1346 patients with chronic HF, it was concluded that both the Gal3 values measured at baseline and the measurement performed 4 months after inclusion were significantly correlated with hospitalization for HF and all-cause mortality. Furthermore, Gal3 levels below 16.2 ng/mL were related with lower admissions for HF [22]. In another study, the CORONA (Controlled Rosuvastatin Multinational Trial in Heart Failure) that included 1329 patients with chronic HF, it was observed that Gal3 values measured at baseline and those measured at 3 months of follow-up were related to increased mortality and rehospitalization for HF [7]. A meta-analysis published in 2017 showed a significant increase in the risk of cardiovascular mortality for each increase in the standard deviation of Gal3 in patients with HF [23].

On the contrary, other studies have not demonstrated the predictive capacity of Gal3 in patients with chronic HF. In the study by Miller et al. [24], which included 180 patients with chronic HF with reduced left ventricular ejection fraction (LVEF) with a two-year follow-up, no relationship was found between Gal3 values and mortality events or heart transplantation after adjusting the results for clinical variables and other biomarkers, such as ST2 and troponin T. When analyzing ST2 and Gal3 as continuous variables, both were independent predictors, which did not occur when they were included separately in the multivariate analysis, where Gal3 lost its predictive value, possibly because Gal3 is influenced by other biomarkers.

Renal function appears to play a key role in determining plasma Gal3 values and appears to influence its predictive value in HF. In HF patients who also have renal failure, Gal3 has a decreased predictive value after adjustment for renal function [25]. These findings could justify the conflicting results in the different studies.

Gal3 is not able to distinguish between patients with HF with reduced or preserved LVEF. However, it is related to diastolic dysfunction severity and ventricular stiffness in patients with HF with preserved LVEF [26]. In this sense, our group has recently published a study including 115 patients with persistent nonvalvular AF, of which 87 (75.65%) presented HF criteria with nonreduced LVEF [27]. Differences in biomarkers (Gal3, ST2, urate, ultrasensitive troponin T, NT-proBNP, fibrinogen, and C-reactive protein (CRP)) were analyzed between patients with and without HF. The only biomarker that was related to the presence of HF was NT-proBNP. No relationship was found between Gal3 and ST2 with the presence of HF. In addition, the same study analyzed the differences in biomarkers between patients with HF with preserved LVEF and HF with mid-range LVEF. The only biomarker that was related to the presence of HF with mid-range LVEF was ultrasensitive troponin T. Gal3 and ST2 did not show any relationship with the presence of IC.

Clinical guidelines provide a class IIb recommendation to consider Gal3 measurement as an additional risk factor in HF [28]. However, as it is not a specific cardiac biomarker, and it is not clear which organs are capable of increasing its levels. More studies will be required to provide knowledge of the role of Gal3 in the pathophysiology of HF. Table 1 shows the main studies of Gal3 in HF.

The predictive value of ST2 in HF is on the rise. In fact, the American Heart Association/American College of Cardiology guidelines on the management of HF recommend (class IIb, level of evidence B) the measurement of ST2, in addition to other fibrosis biomarkers in patients with acute HF for a more appropriate stratification [28].

ST2, unlike NT-proBNP, is not influenced by age, body mass index (BMI), renal function, or the etiology of HF [29]. Furthermore, the measurement of ST2 at the time of admission of a patient with acute HF has been shown to be superior to that of NT-proBNP in predicting mortality at one year [30]. In addition, ST2 presents dynamic changes in its concentration in the evolution of HF. Reduction in ST2 values after treatment adjustment for HF decompensation was related to prognosis regardless of NT-proBNP values [31]. 

Aleksova et al. [8] proposed an algorithm for action in patients who consult for dyspnea and who present high levels of natriuretic peptide (NP) depending on the ST2 value. Values of ST2 <35 ng/mL translate an unlikely diagnosis of acute HF, so other causes of increased NP should be sought. Levels between 35 and 70 ng/mL are associated with mild-moderate acute HF and ST2 levels >70 ng/mL are associated with significant activation of the neurohormonal system and cascades of fibrosis, recommending hospitalization and neurohormonal treatment with the goal of a decrease in ST2 of 30% at discharge. Table 2 shows the main studies of ST2 in HF.

## 5. ST2 and Galectin-3 in Atrial Fibrillation

In AF, there is damage and remodeling of the atrial myocardium, which increases the expression and secretion of Gal3, causing the production of an extracellular matrix through the TGF-b/SMAD signaling pathway that causes fibrosis of the atrial myocardium, perpetuating the mechanisms that maintain AF with the consequent increase in Gal3 levels [2].

Gal3 has been associated with the development of AF in numerous studies, as well as its progression, its expression being highest in persistent forms of the disease [7]. Likewise, Gal3 levels have been independently correlated with the extent of fibrosis at the level of the left atrium by magnetic resonance imaging [32]. In a sheep model of tachypacing-induced AF, the effects of Gal3 inhibition during AF progression were tested. The animals treated with a Gal3 inhibitor had significantly longer action potential durations and fewer rotors and wavebreaks during AF, and myocytes had a lower functional expression of inward rectifier K+ channel (Kir2.3) than saline-treated animals. As a conclusion, Gal3 inhibition may be a potential new upstream therapy for the prevention of AF progression [33].

Gal3 has also been studied in patients with persistent AF without structural heart disease undergoing catheter ablation. The patients in whom AF recurred had significantly higher Gal3 values than those who remained in sinus rhythm. This suggests that Gal3 could be a useful marker to identify suitable candidates for pulmonary vein ablation [34].

Furthermore, Gal3 has been associated with an increased thrombotic risk in patients with AF. This can be explained for two reasons. First, it has been previously associated with the presence of various factors that increase thrombotic risk, assessed on the CHA2DS2-VASc scale, such as hypertension and age. In addition, Gal3 has the ability to bind to the von Willebrand factor that is involved in modulating the initial formation of the thrombus [4]. Furthermore, it has been significantly related to the presence of echo contrast in the left atrial appendage and has a significant negative correlation with the velocity of flow emptying in the left atrial appendage as assessed by transesophageal echocardiography [35].

Recently there have been several publications in this regard, with contradictory results. In a recent meta-analysis, which includes 28 studies with more than 10,000 patients, it was shown that those patients in whom sinus rhythm was restored in different ways and remained in sinus rhythm presented lower levels of Gal3 than those in whom AF recurred. [36]. Another study by Begg et al. [37], included 92 patients undergoing pulmonary vein ablation in whom a blood sample was drawn prior to ablation. The relationship of four biomarkers, including Gal3, with the recurrence of AF in the follow-up of 6 months after the procedure was analyzed. None of them were related to the recurrence of AF. These different results may be due to the multitude of processes in which Gal3 is involved. Currently, there are numerous discrepancies between published studies regarding the relationship between Gal3 and the recurrence of AF.

In addition, the usefulness of ST2 is not established in AF. Although significantly higher ST2 values have been evidenced in patients with both persistent and permanent AF compared to subjects in sinus rhythm, no significant differences were found between patients with paroxysmal and persistent AF [38].

The determination of this biomarker could be useful to detect patients with AF originating in the pulmonary veins and who would obtain greater benefit from ablation than those patients with more advanced disease. In this sense, the relationship between ST2 levels and recurrence of AF has been studied in a group of 100 patients with paroxysmal AF who underwent cryoablation of pulmonary veins. For this, peripheral blood samples were collected prior to ablation. The biomarkers they studied were urate, NT-proBNP, high-sensitivity C-reactive protein, and ST2. ST2 was the only biomarker that was independently and significantly related to AF recurrence. This fact is explained by the authors as a form of expression of extensive fibrosis in the left atrium, and these patients obtain less benefit from ablation [39].

A performance algorithm has been described in maintaining sinus rhythm based on ST2 values [40]. This algorithm was based on the hypothesis that elevated ST2 levels translate into excess myocardial fibrosis. Thus, patients with high levels (considering the cut-off point of 35 ng/mL) would not benefit from performing electrical cardioversion and should be evaluated in a specialized consultation to assess pulmonary vein ablation.

In a recent study of our group [41] in which 115 patients with persistent nonvalvular AF who underwent electrical cardioversion were included, seven biomarkers were analyzed (Gal3, ST2, ultrasensitive troponin T, CRP, urate, fibrinogen, and NT-proBNP) at baseline (prior to performing electrical cardioversion) and at the 6-month follow-up, and its possible relationship with electrical recurrence of arrhythmia was evaluated at the 6-month follow-up. The patients were evaluated with a holter-ECG at 3 months, electrocardiogram at 6 months, in addition to other possible eventual records in their clinical history. None of the biomarkers measured at baseline were related to the presence of recurrence at follow-up. However, ST2 and NT-proBNP measured at the 6-month follow-up were related to the presence of recurrence. The authors suggested that this fact may be due to the existence of mechanisms of inflammation and fibrosis in the acute phase of the fall in AF and that these mechanisms diminish over time. In this sense, no relationship was found with the biomarkers measured at baseline, but there was at follow-up. Gal3 did not show a relationship with recurrence at baseline or at follow-up, possibly due to the variability of the processes in which it is involved.

## 6. Conclusions

Gal3 and ST2 have been shown in numerous studies to be promising biomarkers in the prediction of various cardiovascular diseases. However, there are still conflicting data, especially in the case of Gal3. These contradictions could be clarified in future studies that help us expand their clinical utility and adapt it to daily practice.

## Figures and Tables

**Table 1:** Gal3 studies in heart failure.

Authors	Sample Size	Main Findings
Ho, J.E. et al. [6]	3353	Gal3 significantly predicted the development of IC.
Miro, O. et al. [19]	115	Gal3 predicted mortality one month after admission for HF.
Meijers, W.C. et al. [20]	592	Predictor of the absence of events in the 180 days post-discharge after an episode of decompensation of HF.
Demissei, B.G. et al. [21]	1161	Gal3 remains stable over time. No benefit of repeated measurement of Gal3. Gal3 showed no independent relationship with cardiovascular mortality at 180 days.
Anand, I.S. et al. [22]	1650	Elevated Gal3 values were significantly correlated with hospitalization for HF and all-cause mortality.
van der Velde, AR. et al. [7]	1329	Gal3 values measured at baseline and those measured at 3 months follow-up were related to increased mortality and rehospitalization for HF.
Imran, TF. et al. [23]	32,350	Significant increase in the risk of cardiovascular mortality for each increase in the standard deviation of Gal3 in patients with HF.
Miller, WL. et al. [24]	180	No relationship was found between Gal3 values and mortality events or the performance of a heart transplant.
Beltrami, M. et al. [26]	98	Gal3 was not able to distinguish between patients with HF with preserved or reduced LVEF.
Merino-Merino, A. et al. [27]	115	Gal3 was not related with HF in patients with AF.

AF: atrial fibrillation. Gal3: Galectin-3. HF: Heart Failure. LVEF: Left Ventricular Ejection Fraction.

**Table 2:** ST2 studies in heart failure.

Authors	Sample Size	Main Sindings
Bayer-Genis, A. et al. [29]	879	ST2, unlike NT-proBNP, is not influenced by age, BMI, kidney function, or the etiology of HF.
Manzano-Fernandez, S. et al. [30]	447	At the time of admission of a patient with acute HF, it is superior to that of NT-proBNP in predicting mortality at one year.
Bayes-Genis, A. et al. [31]	48	The reduction in ST2 values after treatment adjustment for HF decompensation was related to the prognosis regardless of the NT-proBNP values.

HF: Heart Failure. BMI: Body Mass Index.

## Data Availability

Raw data were generated at University Hospital of Burgos. Derived data supporting the findings of this study are available from the corresponding author J.-A.P.-R. on request.
